# Clinical results after open gluteus medius repair in single-row technique

**DOI:** 10.1186/s40634-022-00483-x

**Published:** 2022-06-11

**Authors:** Alexander Derksen, Eike Lonnemann, Stefan Budde, Onno Becker, Nils Wirries, Marco Haertlé, Henning Windhagen

**Affiliations:** 1 Department of Orthopaedic Surgery at DIAKOVERE Annastift, Hannover Medical School, Anna-von-Borries-Str. 1-7, 30625 Hannover, Germany; 2 Department of Trauma and Orthopaedic Surgery at Evangelisches Klinikum Bethel (EvKB), University Hospital OWL, University of Bielefeld, Am Burgsteig 13, 33617 Bielefeld, Germany

## Abstract

**Purpose:**

The aim of this retrospective study is to present the clinical results of open repair of gluteus medius and gluteus minimus tendon tears using the single-row suture anchor technique.

**Methods:**

This retrospective study included 43 participants after open repair of the abductor tendon of the hip joint using a single-row suture anchor technique. Clinical outcome parameters were assessed by VAS (0–10), gait safety (1 = absolutely safe gait without assistive devices - 10 = gait not possible), SHV (0–100%), mHHS, WOMAC, and gluteal muscle strength status from single-leg stance and against gravity.

**Results:**

Thirty-two female and 11 male subjects with an average age of 65.2 years were included in this study. Overall, a significant decrease in pain (VAS 3.2) and improvement in joint function is observed after a period of 22.3 months. The mHHS reached 61.9 points, WOMAC 28.2, SHV 69.8% and gait stability measured by the VAS reached 3.4. 58.1% of the participants reported not having Trendelenburg, while 4% could not control the single leg stance.

**Conclusions:**

The present study shows that single row repair for open glutueus medius refixation indicates limited clinical results. Although there was an improvement in clinical outcome. The majority of subjects continued to report limiting symptoms.

## Introduction

Based on symptoms, gluteal tendon tears are related to greater trochanteric pain syndrome (GTPS) along with tendinopathies of the tendon, bursitis trochanterica and coxa saltans externa [5]. The incidence of GTPS is 10–25% and females are four times more affected than males [20]. Tears of the gluteus medius tendon can lead to significant functional limitation of the hip joint with gait disturbance due to a limping gait pattern (Trendelenburg limp) [3]. In addition, there are usually typical GTPS symptoms such as chronic lateral hip pain and tenderness over the greater trochanter [25]. Since the first report by Bunker et al. in 1997 with the initial term ‘rotator cuff tears of the hip’, the pathology has attracted increasing attention [3]. In the majority of cases, gluteus tendon lesions occur degeneratively [13]. Howell et al. observed that a tendon lesion was present in 22% of women and 16% of men during hip arthroplasty [13]. Furthermore, iatrogenic causes could be observed. After reaming to treat a femoral neck fracture by Gamma nail, tendon lesions were seen in 27%, and after implantation of hip arthroplasty with a lateral approach, tendon lesions existed significantly more often compared to a direct anterior approach [2, 21]. In addition, Bremer et al. found that an anterior approach was associated less frequently with fatty degeneration of the gluteus medius compared to the lateral approach [2]. Due to different causes leading to GTPS with the same symptoms, the diagnosis of a tendon lesion should be confirmed without doubt with clinical examination, imaging by native x-ray (to exclude bony causes) sonography and/or MRI imaging [4]. In addition, local anesthetic injection into the trochanteric area is recommended to confirm the diagnosis [14]. Surgical treatment is recommended if symptoms persist after nonoperative treatment has been exhausted [26]. Repair of gluteal tendon ruptures has so far shown satisfactory results with improvement of pain and strength with a low complication rate [1]. The aim of this retrospective study is to present the clinical results after open repair of gluteus medius tendon tears in single-row suture anchor technique. We hypothesized that the clinical results show improvement, but residual symptoms.

## Material and methods

This retrospective study evaluates the clinical outcome after open repair of the gluteus medius tendon using the single-row suture anchor technique. In addition, clinical outcomes were compared between subjects with a gluteus medius tear without prior surgery and after hip arthroplasty, as well as considering preoperative muscle status and tear pattern. All subjects in this study reported persistence of symptoms for more than 6 months. Until then, conservative treatments did not provide any relief of the symptoms.

Inclusion criteria were present for Trendelenburg limp, in which the contralateral side of the pelvis tilts downward during stance and/or tenderness over the gluteal insertion. The diagnosis was confirmed by partial- or full-thickness tears. In addition, a local anesthetic was injected into the trochanteric area to prove that the cause of the pain was the tendon lesion.

Exclusion from the study existed only in the event of patient refusal to participate or in the absence of contact information.

Using medical history collected from the hospital information system with preoperative detailed physical examination findings and demographic data (eg. age, sex, laterality, date of surgery, and presence of total hip arthroplasty) were evaluated. The preoperative quantification of the tendon lesion was performed using the MRIs of the pelvis from the clinic’s internal Picture Archieving and Communications System. Tear pattern was classified into a full thickness lesion or a partial-thickness tear. The muscle condition was assessed in comparison to the opposite side, taking into account fatty degeneration (classified according to the Goutallier-Fuchs [11]) as well as tendon retraction (yes/no). When images were no longer available, this information was taken from the preoperative and intraoperative medical reports.

In the study subjects, six cases had a full-thickness tear pattern, three of which had retraction of the tendon. In 37 cases, a partial-thickness tear pattern was present. There was muscle atrophy in nine cases, of which three had 1° and one had 2° fatty infiltration. In none of the cases was a 3° or 4° fatty infiltration found. Furthermore, in five cases there was a low-grade calcification in the region of the tendon insertion.

Using patient self-administered questionnaires, the following variables were retrospectively reviewed for preoperative and current status:

- Visual analog scale for pain (0 is considered no pain - 10 is considered worst pain; VAS)
- Subjectively assessed gait safety with evaluation based on VAS (0 = absolutely safe gait; 10 independent gait not possible; VGS)
- Subjective Hip Value (0–100%; SHV) [18]
- Modified Harris Hip Score (mHHS) [12]
- Western Ontario and McMaster Universities Osteoarthritis Index (WOMAC) in German version [23]. After adjustment, 100 points are achievable, representing the worst possible outcome.

Current status:

- Detection of previous hip arthroplasty surgeries.
- Functional status of the affected gluteal musculature evaluating the presence of a Trendelenburg sign (y/n), possibility of a safe one-legged stance (y/n), and abduction ability of the hip against gravity (not possible, 1–10 times, > 10 times).

This study was approved by the institutional review board (No. 3660–2017).

### Surgical technique

**Fig. 1:**
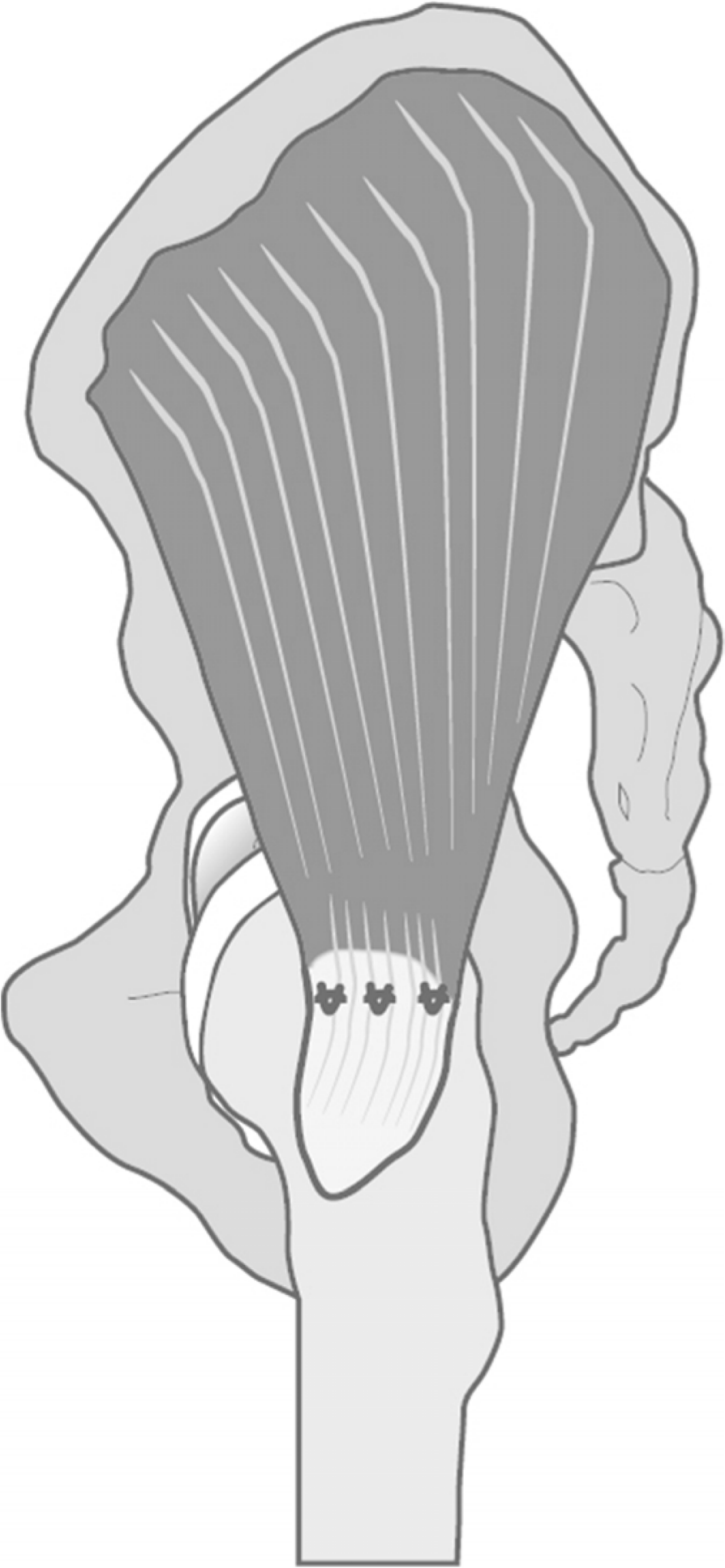
Visualization of the repaired gluteus medius tendon using single-row suture anchor technique

All subjects were operated on by a senior surgeon. The tendon was repaired using a single-row suture anchor technique with open surgical approach [19]. The patient was positioned in the lateral position. The leg was positioned on a TRIMANO FORTIS® Support Arm (Arthrex Inc., Naples, FL) and placed in neutral position of the hip joint. A 4–5 cm incision in the midline from the trochanter was made, the IT band was presented and dissected along the fibers. In the presence of trochanteric bursitis, it was excised while protecting the tendon. The tendon was inspected and the rupture identified, which could be optimally performed through the open approach. In case of adhesions, they were debrided. To visualize articular tendon defects, the tendon was split along the fibers in the midline. This was followed by mobilization of all tendon parts and debrading by removal of adhesions until the tendon could be placed in the anatomical position at the footprint on the greater trochanter, in neutral position of the hip joint, under a low residual tension. For repair, the limb was positioned in 20°- 25° abduction using the TRIMANO FORTIS® Support Arm. The entry points for the anchors were prepared with a 4.5 mm punch and tap for Corkscrew anchors (Arthrex Inc., Naples, FL). The single-row suture anchor technique on the gluteus medius tendon was performed with three 4.5-mm Bio-Composite Corkscrew anchors (Arthrex Inc., Naples, FL) considering the posterior and anterior border of the lateral facet of the footprint (Fig. 1). The repair was tested in neutral position of the hip joint, following in 90° flexion and 20° extension to ensure stability. Afterwards, the wound was irrigated with normal saline. Finally, the IT band was closed by single sutures, followed by subcutaneous sutures and skin clamps.

### Post treatment

All patients received the same postoperative treatment regimen. Partial weight-bearing with plantar to floor contact without additional weight-bearing was recommended for 6 weeks. Adduction above neutral and active abduction were not allowed for 8 weeks. Subsequently, pain-adapted weight-bearing was allowed.

### Statistical analysis

Data were evaluated retrospectively. The statistical analysis was performed using IBM SPSS Statistics for Windows (Version 24; IBM Corp., Armonk, NY). The chosen significance level for all variables examined was *p* < 0.05. For analysis of the independent ordinal variables (Visual analogue scale, Paris Hip Score and WMOMAC Score) between the two groups (with and without hip arthroplasty) the Mann- Whitney- U- Test was used. Examining the categorial variables (such as physiotherapy, necessity of further surgery, positive trendelenburg sign, etc.) contingency tables were used. For investigation of changes from preoperatively to postoperatively of the depended ordinal variables the Wilcoxon test was used.

## Results

From August 2014 to December 2017, 60 patients were treated with a single-row suture anchor technique of gluteus tendon rupture. Of the 17 patients who could not be included, 2 had died in the period leading up to the study, 7 declined to participate, and contacting for 8 participants was not possible because of a change in residential address.

Forty-three patients could be included as subjects in this study with a mean follow-up of 22.8 months (± 9.7; 9–46 months): 32 female (74.4%) and 11 male (25.6%) subjects, with a mean age of 65.2 years (± 11.3; 41.3–85.1 years). Tendon repair was performed on the right side in 24 cases (55.8%) and on the left side in 19 cases (44.2%).

The evaluated PROMS showed a significant improvement in outcomes at the time of study (post-op) compared with the preoperative condition (Table 1).

**Table 1:** Results of PROMS with mean ± standard deviation (minimum - maximum)

PROMS	Pain mHHS	Gait mHHS	Activities mHHS	mHHS total	Pain WOMAC	Function WOMAC	Stiffness WOMAC	WOMAC total
**Pre-OP**	16.7 ± 10.9 (10–44)	18.0 ± 8.3 (2–33)	8.1 ± 3.1 (3–14)	**42.4 ± 19.7** **(20–87)**	45.4 ± 29.6 (1.8–93)	44.0 ± 30.2 (0.5–94.1)	43.6 ± 31.2 (0–98)	**44.3 ± 29.3** **(0.8–90.1)**
**Post-OP**	26.2 ± 13.1 (10–44)	24.6 ± 8.1 (7–33)	10.5 ± 3.1 (3–14)	**61.9 ± 21.3** **(26–91)**	29.0 ± 28.2 (0–99)	27.7 ± 26.2 (0.1–90.1)	30.9 ± 26.2 (0–81)	**28.2 ± (25.9)** **(0.1–85.7)**
**p- value**	*p* < .001	*p* < .001	*p* < .001	***p*** **< .001**	p .003	p .002	p .02	**p .003**

*mHHS* modified Harris Hip Score and, *WOMAC* Western Ontario and McMaster Universities Osteoarthritis Index comparing preoperative and condition (Pre-op) at the time of examination (Post-op), *p* = statistical significance at significance level < 0.05

Pain intensity measured using the VAS was 7.7 (± 1.7; 3.3–10) preoperatively and 3.6 (± 3.2; 0–9.3) postoperatively, thus decreasing by an average of 4.1 (*p* = .001). Gait safety measured using the VAS was rated with 3.4 (± 3.2; 0–7) and preoperatively with 7.2 (± 2.2; 5–9). General hip joint health as measured by SHV was 69.8% (± 28.8%; 10–100%) befor surgery and 46.2% (± 24.6%; 0–80%) (*p* < .001) after surgery.

### Functional status of the gluteal muscle

The single-leg stance was reported after surgery as safely feasible and without a Trendelenburg sign in 25 subjects (58.1%) and as unsafe by 18 (41.9%), whereas 16 (37.2%) of those continued to report a Trendelenburg sign and 2 (4.7%) were unable to perform the single-leg stand at all due to a risk of falling in the absence of upper body stabilization. Testing of hip joint abduction force from the lateral position against gravity resulted in no limitation with > 10 possible repetitions in 21 (48.8%) participants. 19 (44.2%) achieved 1–10 repetitions and 3 (6.9%) reported being unable to perform the exercise due to lack of strength.

Clinical outcomes were compared between subjects with a gluteal tendon lesion without previous hip joint surgery (*n* = 29; 67.4%) and after total hip arthroplasty (*n* = 14; 32.6%). In these cases, hip arthroplasty was performed 6.9 years (± 7.1; 1–22 years) earlier. The parameters did not reveal significant differences or systematic trends in outcomes between the two groups.

If the clinical results, taking into account the preoperative findings between partial- and full-thickness tear pattern or muscle atrophy, fatty infiltration, tendon retraction and calcification of the trochanteric region compared to without, there are no statistically significant results (*p* > 0.05).

## Discussion

While the gluteal tendon lesion disease received little attention and was relatively unknown until a few years ago, it is now receiving an increasing level of awareness [6, 27]. In 2006, Cormier et al. reported that only 55% of orthopedic surgeons in France were aware of such a disease and that 13% had never seen this disease pattern in their patients [6]. In contrast, in a recent survey by Zhu et al. in New Zealand, 90% of orthopaedic surgeons reported knowing the disease [27]. This is probably caused by an increasing number of publications over the last few years showing an increase interest in the disease pattern [1, 9, 16]. Improved pain and functional outcomes are reported in the majority of publications (Table 1).

This retrospective study presents the clinical outcomes of 43 patients, at an average of 22.8 months after open single-row suture anchor technique of the gluteus tendon due to a defect. Overall, it can be concluded that patients in this study demonstrate pain relief and improved hip joint function. However, it is critical to note that our study shows poorer clinical outcomes compared to most of the other studies as shown in Table 2.

**Table 2:** Comparative studies

Author	n	Mean Follow-up	mHHS	VAS	Repair technique
Our survey	43	22.8	42.4 / 61.9	7.7 / 3.6	op. / sr
Fearon et al. [10]	24	22	n / 67.2	8,5 / 1	op. / to
Davies et al. [7]	22	> 12	53 / 88	X	op. / to
DeFroda et al. [8]	31	6	40.1 / 60.9	X	op. / dr
Nazal et al. [22]	15	31.2	54.2 / 82.9	5.4 / 2.4	end. / dr
Taunaht et al. [24]	22	31.7	33.7 / 80.2	7.2 / 3.2	end. / dr

*number of patients* n, Mean follow-up in months, *mHHS* modified Harris Hip Score (pre/post-operative), *op.* open, *end.* endoscopic, *sr* single-row, *to* transosseous, *dr* double-row, *x* not comparable with VAS

We assume that the reason for this is that the single-row suture anchor technique resulted in insufficient stabilization and a lower overlap of the tendon attachment, so that the tendon may not have been able to grow in sufficiently.

Biomechanical analysis comparing single-row suture versus double-row suture pointed out that the double-row suture anchor technique can result in a larger footprint coverage leading to a higher stability [15, 17].

This finding may be related to a higher healing success. As a result of these observations, we have introduced the double-row suture anchor technique as current standard in our clinic. Because of the retrospective study design, imaging control of refixation success by MRI had not been performed, so this theory could not be verified. In a prospective study, we are currently reviewing the clinical results and monitoring the success status of the refixation in MRI controls.

The present clinical results, taking into account the preoperative muscle and tendon status, did not show statistically significant differences. Due to the small subgroup size, the results can only be considered with limitations.

The publication by Thaunat et al. showed that fatty muscle degeneration has an influence on clinical outcome [24]. However, only cases without prior surgical intervention were reviewed and there was a significantly higher proportion of patients with fatty degeneration (14 of 22) with a higher degree of fatty degeneration (four subjects with more than 2° of fatty infiltration). In this study, only four participants showed fatty infiltration and none were found to have 3 or 4 degrees of expression. Therefore, the comparability to our study is limited.

Alpaugh et al. reported in their review of 8 studies that re-tears occurred in 9% after open refixation, compared to no cases reported after endoscopic Refixation [1]. This lead to the interpretation that the open surgical technique increases complications.

However, it is important to consider that there is a difference in number of participants (Total from all studies = 135 open vs. 39 endoscopic). Therefore, the studies reflect a significantly larger group of participants after open repair. It cannot be ruled out that the poorer results in our study are related to the surgical technique used, because a re-tear review was not performed and no comparison group after endoscopic treatment was available.

One of the main limitations is the retrospective study design and the lack of postoperative MRI imaging to verify the repair status, which means that re-rupture cannot be excluded. Thus, a comparison between groups with failure and successful repair is not possible.

Although the clinical examination was assessed by means of patient self-administered questionnaires, which can be seen as a deficiency. All participants responded that they had no further questions in this regard, this indicates a good understanding regarding the question. However, in this study, a comparatively large study population is available after open single-row suture anchor technique. Also, there was no selection or exclusion of subjects in the study population. Therefore, we assume that these data are relevant for subsequent studies with higher levels of evidence.

## Conclusion

The present study shows an improvement in clinical outcome, the majority of subjects continued to report limiting symptoms. However, a part of the patients presents with persistent complaints. It may be that a single-row refixation is insufficient and leads to reduced healing. Further studies with comparison groups and a prospective study design as well as imaging review are needed to validate the present results.
